# Promoting tomato resilience: effects of ascorbic acid and sulfur-treated biochar in saline and non-saline cultivation environments

**DOI:** 10.1186/s12870-024-05734-w

**Published:** 2024-11-08

**Authors:** Muhammad Ikram, Asif Minhas, Arwa A. AL-Huqail, Adel M. Ghoneim, Sammina Mahmood, Esawy Mahmoud, Maryam Tahira, Muhammad Mehran, Muhammad Faizan Khurram Maqsood, Abdul Rauf, Waqar Ali

**Affiliations:** 1 Department of Agronomy, Faculty of Agricultural Science’s and Technology, Bahauddin Zakariya University , Multan, Pakistan; 2 MOA Key Laboratory of Crop Ecophysiology and Farming System in the Middle Reaches of the Yangtze River, College of Plant Science & Technology, Huazhong Agricultural University, Wuhan, 430070 China; 3 Department of Biology, College of Science, Princess Nourah bint Abdulrahman University, P.O. Box 84428, Riyadh, 11671 Saudi Arabia; 4 Agricultural Research Center, Field Crops Research Institute, Giza, 12112 Egypt; 5 Department of Botany, Division of Science and Technology, University of Education, Lahore, Pakistan; 6 Soil and Water Department, Faculty of Agriculture, Tanta University, Tanta, 31511 Egypt; 7 National Key Laboratory of Horticultural Plant Biology, Ministry of Education, Wuhan, Hubei 430070 China; 8 Key Laboratory of Arable Land Conservation (Middle and Lower Reaches of Yangtze River), Ministry of Agriculture and Rural Affairs, Huazhong Agricultural University, Wuhan, 430070 China; 9 Center for Eco-Environment Restoration Engineering of Hainan Province, School of Ecology, Hainan University, Haikou, 570228 China

**Keywords:** Tomatoes, Salinity, Ascorbic acid, Sulphur treated biochar, Antioxidants, Macronutrients

## Abstract

The resilience of tomato plants under different cultivation environments, particularly saline and non-saline conditions, was investigated by applying various treatments, including 0.5% Ascorbic Acid (AsA) and 1% Sulphur-treated Biochar (BS). The study evaluated parameters such as fruit length, diameter, yield per plant and pot, Total Soluble Solids (TSS) content, chlorophyll content, electrolyte leakage, enzyme activities (Superoxide Dismutase - SOD, Peroxidase - POD, Catalase - CAT), and nutrient content (Nitrogen - N%, Phosphorus - P%, Potassium - K%). Under saline conditions, significant enhancements were observed in fruit characteristics and yield metrics with the application of AsA and BS individually, with the combined treatment yielding the most substantial improvements. Notably, AsA and BS treatments exhibited varying effects on TSS levels, chlorophyll content, electrolyte leakage, and enzyme activities, with the combination treatment consistently demonstrating superior outcomes. Additionally, nutrient content analysis revealed notable increases, particularly under non-saline conditions, with the combined treatment showcasing the most significant enhancements. Overall, the study underscores the potential of AsA and BS treatments in promoting tomato resilience, offering insights into their synergistic effects on multiple physiological and biochemical parameters crucial for plant growth and productivity.

## Introduction

Tomato (*Solanum lycopersicum*) is one of the most economically significant vegetable crops worldwide, valued for its nutritional content, culinary versatility, and widespread culinary use. However, tomato cultivation faces numerous challenges, including abiotic stresses such as salinity, which can significantly reduce yields and quality [1, 2]. Tomatoes are considered moderately sensitive to salinity, with yield reductions beginning at an electrical conductivity (EC) level of 2.5 dS/m. Research indicates that for every 1 dS/m increase beyond this threshold, tomato yields typically decrease by 9–10%. For example, under moderate salinity conditions (2.5–4 dS/m), yield losses can range from 10 to 20%, whereas under high salinity levels (greater than 4 dS/m), reductions can reach up to 50% depending on the tomato cultivar and the duration of exposure. The impact on yield is directly tied to salinity’s effect on water uptake and ion toxicity, which disrupts essential physiological processes, including photosynthesis and nutrient absorption. In addition to yield loss, salinity stress also affects fruit quality, reducing fruit size, altering the sugar-acid balance, and, in severe cases, compromising marketability. While some salinity can enhance Total Soluble Solids (TSS), excessive levels lead to small, unmarketable fruit with poor texture and appearance. These quantified impacts provide a clearer understanding of the severity of the problem, offering insight into why salinity management is crucial in tomato production. Salinity stress, resulting from the accumulation of soluble salts in the soil, disrupts plant water balance, ion uptake, and cellular metabolism, leading to osmotic stress, ion toxicity, and oxidative damage. Saline soil is a significant element that restricts crop production. By 2050, it is anticipated that 50% of the world’s arable land will be affected by salt stress [3]. Sodium chloride stress, common salt stress, significantly harms crop growth through osmotic stress and particular ion toxicities. Salt stress can affect crops at every growth stage, from germination to maturity. Germination and early seedling growth phases are more salt-sensitive in most plant species than other growth stages [4].

In recent years, there has been growing interest in developing sustainable agricultural practices to enhance crop resilience and productivity under adverse environmental conditions. Among the various strategies investigated, applying ascorbic acid (vitamin C) and sulfur-treated biochar has shown promising results in improving plant tolerance to salinity stress and enhancing overall growth and yield.

Ascorbic acid (AsA), also known as vitamin C, plays a crucial role in enhancing plant resilience under stress conditions like salinity by functioning through several key mechanisms. As a potent antioxidant, AsA scavenges reactive oxygen species (ROS), preventing oxidative damage to cellular components, and participates in the ascorbate-glutathione cycle to maintain redox balance. It also regulates the activity of antioxidant enzymes such as Superoxide Dismutase (SOD), Peroxidase (POD), and Catalase (CAT), further aiding in ROS detoxification [5–7]. AsA is essential for maintaining photosynthetic efficiency by protecting photosystem II from oxidative damage, ensuring energy production and safeguarding the Calvin cycle [8]. Additionally, it helps regulate ion homeostasis, particularly by balancing sodium (Na⁺) and potassium (K⁺) levels under salinity stress, thereby sustaining cell turgor and osmotic balance. AsA modulates the expression of stress-responsive genes and influences hormonal pathways, including abscisic acid (ABA), which is key to stress response regulation [9]. Moreover, AsA strengthens plant cell walls by promoting the biosynthesis of structural components, enhancing the overall stability and stress tolerance of the plant. These mechanisms collectively enhance the physiological and biochemical resilience of plants, particularly under salinity stress [10]. At the same time, sulfur-treated biochar improves soil structure, nutrient availability, and microbial activity, thereby promoting plant growth and stress tolerance [11]. This study aims to investigate the effects of ascorbic acid and sulfur-treated biochar on tomato resilience under both saline and non-saline cultivation environments. By elucidating the physiological and biochemical mechanisms underlying the interactions between these treatments and salinity stress, we seek to provide insights into potential strategies for enhancing tomato productivity and sustainability in saline-affected regions. Additionally, we aim to assess the feasibility and economic viability of implementing these strategies in commercial tomato production systems, considering factors such as cost-effectiveness, environmental impact, and long-term sustainability.

The study evaluates the practicality and efficiency of using saline water for irrigating tomato plants (*Solanum lycopersicum L*.). Tomatoes are fairly resilient to salt stress. A recent analysis has examined the effects of salinity on various aspects of tomato plants, including morphology, physiology, biochemistry, yield, fruit quality, and gene expression [12, 13]. To assess the possible use of biochar to mitigate salinity-related difficulties in tomato cultivation. The study evaluated the impact of salt stress alone or combined with biochar added to the growth medium on plant growth, chlorophyll content, antioxidant response activation, and nutritional status. Ascorbic acid, a well-known antioxidant, plays a critical role in enhancing plant tolerance to various abiotic stresses, including salinity. Several studies have demonstrated the effectiveness of AsA in mitigating the adverse effects of salinity stress. Researchers has reported that exogenous application of AsA improved salinity tolerance in rice by enhancing antioxidant enzyme activities, reducing oxidative damage, and maintaining higher levels of chlorophyll and photosynthetic efficiency. Similarly, studies on wheat and maize have shown that AsA helps in scavenging reactive oxygen species (ROS) and promotes better ion homeostasis under salt stress conditions [14, 15]. Research on tomatoes under salinity stress has also highlighted the positive effects of AsA. It is found that AsA-treated tomato plants showed improved growth, photosynthesis, and yield under salinity stress due to the enhancement of antioxidant defense systems. AsA’s role in maintaining membrane stability, reducing lipid peroxidation, and regulating sodium and potassium balance has been repeatedly noted in several studies [16, 17]. However, despite the progress, there are limited comprehensive studies comparing the efficacy of AsA under combined treatment conditions, such as in combination with biochar, under salinity stress [18]. This study aims to investigate how 0.5% Ascorbic Acid (AsA) and 1% Sulfur-treated Biochar(BS) affect tomato plants (*Solanum lycopersicum*) under saline and non-saline conditions. Specifically, it explores their impact on growth, yield, fruit quality, and antioxidant enzyme activities, hypothesizing that the combined treatment will offer superior protection against salinity stress. We also assess the feasibility and economic viability of these treatments in commercial tomato production. It is expected that AsA and BS, especially when combined, will improve plant resilience, mitigate the adverse effects of salinity, and enhance productivity.

## Materials and methods

### Experimental site

The evaluation was carried out under pot experiment in the research area of the Faculty of Agricultural Sciences and Technology, Bahauddin Zakariya University Multan (Punjab) Pakistan, which is situated at geographical coordinates 30°15′49″N &71°30′35″E. The experimental design applied completely randomized design (CRD) with four replications for the study execution year, i.e., 2023. The physiochemical properties of soil and irrigation water are given in Table 1.

**Table 1:** Pre-experimental soil and irrigation characteristics

Soil	Values	References	Irrigation	Values	References *ences*
pH	8.19	[19]	pH	7.26	[20]
EC*e* (dS/m)	2.42	[21]	EC (µS/cm)	387	
SOM (%)	0.60	[22]	Carbonates (meq./L)	0.00	
TN (%)	0.03	[23]	Bicarbonates (meq./L)	4.69	
AP (µg/g)	6.27	[24]	Chloride (meq./L)	0.05	
EK (µg/g)	127	[25]	Ca + Mg (meq./L)	2.69	
ENa (µg/g)	116	[26]	Sodium (mg/L)	106	
Texture	Clay Loam	[27]			

TN = Total Nitrogen; AP = Available Phosphorus; EK = Extractable Potassium; ENa = Extractable Sodium

### Treatment application

To investigate the effects of Ascorbic Acid (AsA) and Sulphur-treated Biochar (BS) on tomato resilience under saline (6.52 dS/m) and non-saline (1.38 dS/m) growth conditions, a detailed experimental setup was designed. The soil used in the experiment was a loamy mix, with an initial pH of 6.8, an organic matter content of 2.5%, and a baseline electrical conductivity (EC) of 1.38 dS/m in non-saline conditions. Salinity was adjusted using sodium chloride (NaCl) to achieve the desired EC level of 6.52 dS/m for the saline treatments. The soil was mixed thoroughly with 1% sulfur-treated biochar (BS) by weight prior to planting.

Ascorbic Acid (AsA) solutions at a concentration of 0.5% were prepared by dissolving Ascorbic Acid powder in distilled water, and this solution was applied as a foliar spray to the treated plants. Irrigation was applied every three days, using 500 ml of water per pot, ensuring that the saline treatments received water with the specified salt concentration to maintain consistent EC levels. Non-saline treatments were irrigated with distilled water.

### Pot preparation and sowing

Tomato seeds were sown in plastic pots of 20 kg filled with a standardized soil mixture. Before sowing, the saline soil pots were mixed with the 1% BS, and upon germination and seedling establishment, the tomato plants were subjected to 0.5% AsA spray. Plants were grown in a controlled greenhouse environment, with a temperature of 25 °C during the day and 18 °C at night, 60% relative humidity, and 14 h of light per day. Control setup: In the non-saline group, no additional salt was added, and plants were irrigated with non-saline water. The same amounts of biochar and AsA treatments were applied as in the saline setup to ensure consistency across all treatment groups.

### Collecting, sterilization, and sowing of seeds

Tomato seeds of FM-9 variety (procured from an approved seed merchant licensed by the Government of Punjab, Pakistan) were used in this study. The selected seeds were subjected to a rigorous surface sterilization protocol (applied before sowing). The seeds were rinsed three times with 95% ethanol after exposure to the 5% sodium hypochlorite solution. The seeds were then further washed four times with sterilized deionized water to remove any residues of the sterilizing agents. Twenty seeds were sown per pot filled with 5 kg soil. After germination, thinning was achieved at 10 seedlings per pot (Ahmad et al., 2014).

### Harvesting and data collection

A total of 2 harvestings were performed. The first harvesting includes the collection of fresh leaves after 50 days of transplantation (at the vegetative stage) for the analysis of chlorophyll contents, proline, antioxidants, and other biochemical analysis [19]. However, after 85 days of transplantation, tomato picking started. For the assessment of yield, a total of 8 picks were taken [19]. A total of 8 picks were taken to assess yield data. The study encompassed the examination of various parameters, including the total plant dry weight (g/plant), plant height (cm), the number of primary branches per plant, fruit length (cm), fruit girth (cm), fruit yield (kg/plant), and the assessment of chlorophyll and proline content. Fruit length and diameter were measured by Vernier caliper, and fruit yield per pot was recorded at harvest. Total Soluble Solids (TSS) content was measured using a refractometer. Chlorophyll content was determined using a SPAD meter. The quantification of SOD, POD, and CAT activities was performed using immunosorbent assay kits obtained from Keep Biotechnology Co., Ltd. in Jiangsu, China.

### Irrigation

Irrigation in each pot was monitored and adjusted as needed with electrical meters (ADVANCED™; 4 in 1 Soil Meter, China). Daily observations were done to ensure that the moisture level was maintained at wet; on the instrument scale, this corresponds to 70% of field capacity in soil.

### Electrolyte leakage

Electrolyte leakage of plants was determined by incubating one leaf in 20 ml ddH2O in darkness for hours). The samples were then vortexed, and initial electrical conductivity was measured with the help of a conductivity meter. Samples were then autoclaved for 15 min at 60 o C. The samples were then cooled to room temperature, and their conductivity was determined. The electrolyte leakage (µS/cm) was calculated according to the following equation [20]

$$\eqalign{(EL\% ) & = {\rm{ }} & \cr {\rm{Initail electrical conductivity }} \div {\rm{ }} \cr {\rm{final electrical conductivity }} \times 100 \cr}$$

### Macronutrient legislation

Nitrogen, phosphorous, and potassium in plant samples were determined, as explained by the ICARDA manual 3rd edition [21].

### Statistical analysis

The data were analyzed using conventional statistical techniques, namely the two-way ANOVA, to determine treatment significance. The Tukey test was applied for the paired comparisons, with a significance level of *p* ≤ 0.05. OriginPro Software was used for the Pearson correlations [22].

## Results

### Fruit length (cm), Fruit diameter, Fruit plant− 1, and Yield Pot–1 (kg)

**Fig. 1:**
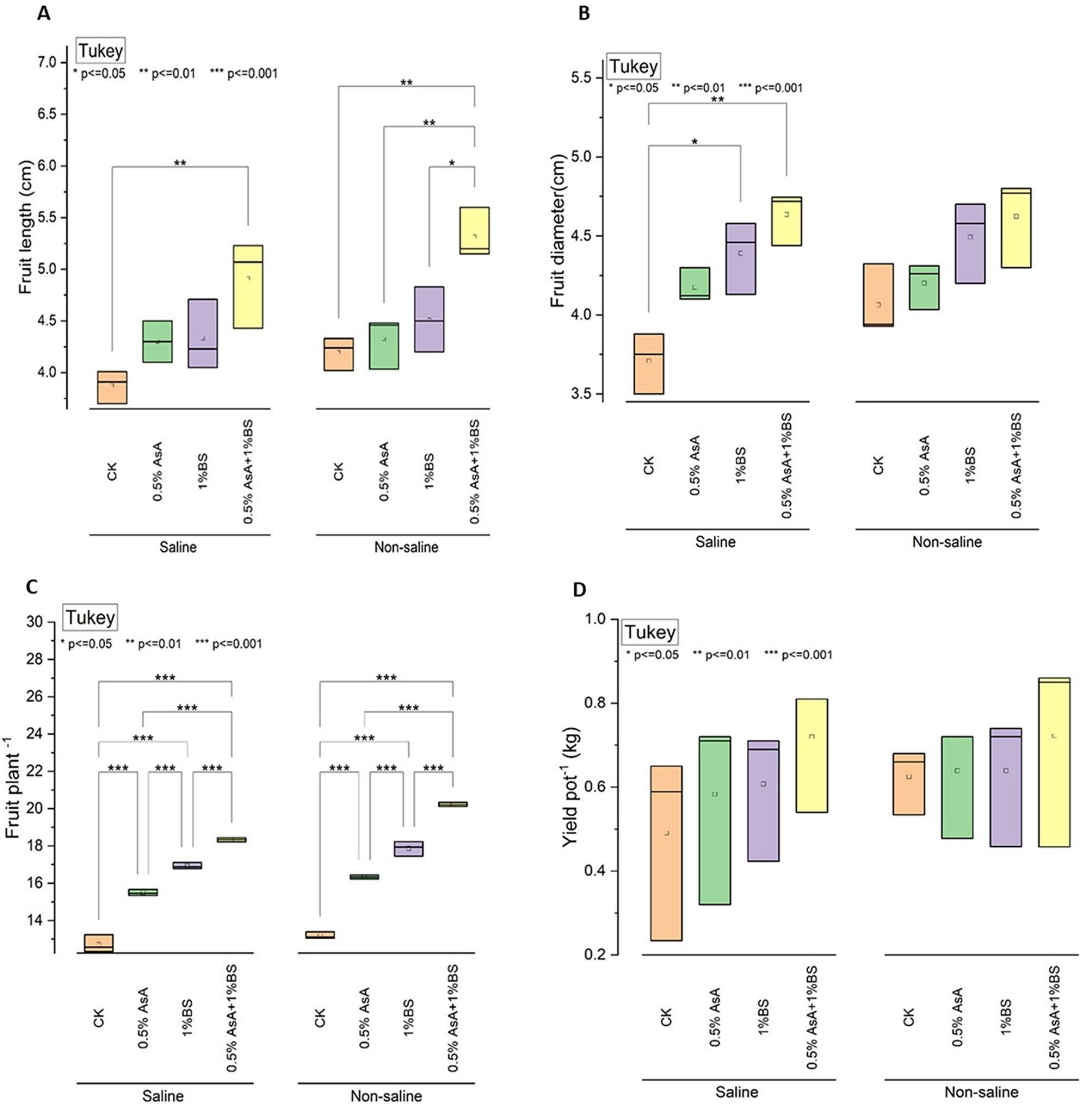
Effect of applied treatments of ascorbic acid (AsA) and sulfur-treated biochar (BS) on the fruit length (cm) (**A**), fruit diameter(cm) (**B**), Fruit plant^− 1^ (**C**), Yield Pot–1 (kg) (**D**)of tomato plants under saline and non-saline growth conditions. The asterisk (*) on the boxplot shows the significant difference among the treatments based on Tukey’s HSD test at *p* ≤ 0.05, 0.01, and 0.001 level

Under saline conditions, treatment with 0.5% Ascorbic Acid (AsA) resulted in an 11.05% increase in fruit length compared to the control (CK), while 1% Sulphur-treated Biochar (BS) led to a 12.63% increase. The combined treatment of 0.5% AsA and 1% BS exhibited the most significant enhancement, showing a 26.73% increase in fruit length relative to CK (Fig. 1A). In contrast, under non-saline conditions, 0.5% AsA led to a 2.86% increase, 1% BS resulted in a 7.38% increase, and the combined treatment showed a 26.67% increase compared to CK. For fruit diameter, under saline conditions, 0.5% AsA resulted in a 12.43% increase, 1% BS led to an 18.10% increase, and the combined treatment achieved a 24.59% increase compared to CK. In non-saline conditions, 0.5% AsA led to a 3.45% increase, 1% BS resulted in a 10.15% increase, and the combined treatment showed a 13.30% increase in fruit diameter compared to CK (Fig. 1B).

Regarding fruit yield per plant, under saline conditions, 0.5% AsA increased yield by 21.7%, and 1% BS resulted in a 33.2% increase. The combination of 0.5% AsA and 1% BS demonstrated the most pronounced effect, with a 44.2% increase in yield compared to CK. Under non-saline conditions, 0.5% AsA led to a 23.8% increase, 1% BS resulted in a 35.5% increase, and the combined treatment achieved the highest enhancement with a 53.3% increase compared to CK (Fig. 1C).

For yield per pot, under saline conditions, 0.5% AsA resulted in an 18.4% increase, and 1% BS led to a 24.5% increase. The combined treatment of 0.5% AsA and 1% BS showed the most substantial rise, with a 47.4% increase in yield per pot compared to CK. Under non-saline conditions, adding 0.5% AsA resulted in a marginal rise of 3.2%, while 1% BS showed no significant difference. The combined treatment of 0.5% AsA and 1% BS did not provide additional benefits compared to the individual treatments (Fig. 1D).

### Total soluble solids, chlorophyll content, electrolyte leakage, and SOD

**Fig. 2:**
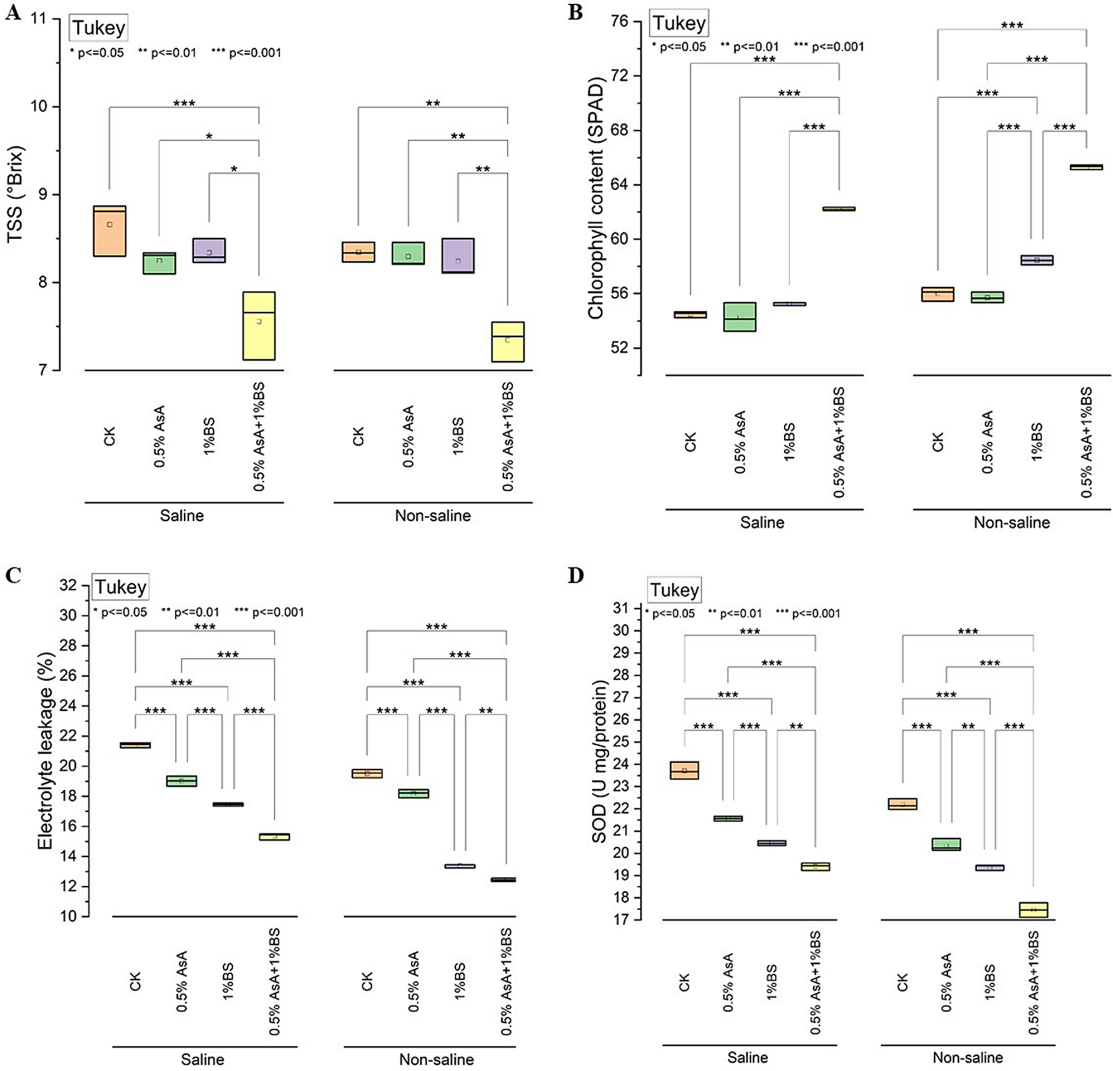
Effect of applied treatments of ascorbic acid (AsA) and sulfur-treated biochar (BS) on the total soluble solids (°Brix) (**A**), Chlorophyll (**SPAD**), Electrolyte leakage (**%**) (**C**), SOD(U/mg) **D** of tomato plants under saline and non-saline growth conditions. The asterisk (*) on the boxplot shows the significant difference among the treatments based on Tukey’s HSD test at *p* ≤ 0.05, 0.01, and 0.001 level

The Total Soluble Solids (TSS) content, measured in degrees Brix (°Brix), of tomato fruits under different growth conditions and treatments revealed distinct patterns. Under saline conditions, the control (CK) exhibited a TSS level of 8.66 °Brix. Treatment with 0.5% Ascorbic Acid (AsA) slightly decreased to 8.25 °Brix, while 1% Sulfur-treated Biochar (BS) led to a decrease to 8.34 °Brix. The combined treatment of 0.5% AsA and 1% BS showed the most significant reduction, with a TSS level of 7.56 °Brix compared to CK (Fig. 2A). In contrast, under non-saline conditions, CK displayed a TSS level of 8.34 °Brix. Applying 0.5% AsA and 1% BS resulted in minimal decreases to 8.30 °Brix and 8.24 °Brix, respectively. The combined treatment of 0.5% AsA and 1% BS exhibited a more substantial decrease, with a TSS level of 7.35 °Brix compared to CK.

For chlorophyll content, measured in SPAD units, CK exhibited 54.48 SPAD units under saline conditions. Application of 0.5% AsA and 1% BS resulted in slight decreases to 54.24 SPAD units and 55.27 SPAD units, respectively. Remarkably, the combined treatment of 0.5% AsA with 1% BS showed the most significant increase, measuring 62.21 SPAD units (Fig. 2B). In non-saline conditions, CK displayed a chlorophyll content of 56.01 SPAD units. Application of 0.5% AsA and 1% BS resulted in relatively minor changes to 55.71 SPAD units and 58.45 SPAD units, respectively. The combined treatment of 0.5% AsA with 1% BS showed a remarkable increase, measuring 65.30 SPAD units compared to CK.

Analysis of electrolyte leakage, represented as a percentage, under saline conditions, CK exhibited an electrolyte leakage of 21.41%. Treatment with 0.5% AsA decreased electrolyte leakage to 19.01%, while 1% BS decreased to 17.45%. The combined treatment of 0.5% AsA and 1% BS showed the most significant reduction, with an electrolyte leakage of 15.35% compared to CK (Fig. 2C). In non-saline conditions, CK displayed an electrolyte leakage of 19.52%. Applying 0.5%, AsA decreased leakage to 18.19%, while 1% BS was reduced to 13.38%.

For Superoxide Dismutase (SOD) activity, measured in units per milligram of protein, under saline conditions, CK exhibited a SOD activity of 23.71 units/mg protein. Treatment with 0.5% AsA decreased SOD activity to 21.56 units/mg protein, and 1% BS resulted in a decrease to 20.45 units/mg protein. The combined treatment of 0.5% AsA with 1% BS showed the most significant reduction, with a SOD activity of 19.41 units/mg protein (Fig. 2D). In non-saline conditions, CK displayed a SOD activity of 22.19 units/mg protein. Application of 0.5% AsA decreased SOD activity to 20.34 units/mg protein, and 1% BS resulted in a reduction to 19.38 units/mg protein. The combined treatment of 0.5% AsA with 1% BS exhibited the most substantial reduction, measuring 17.45 units/mg protein compared to CK.

### Peroxidase(POD), CAT, Nitrogen(%),Phosphorus(%)

**Fig. 3:**
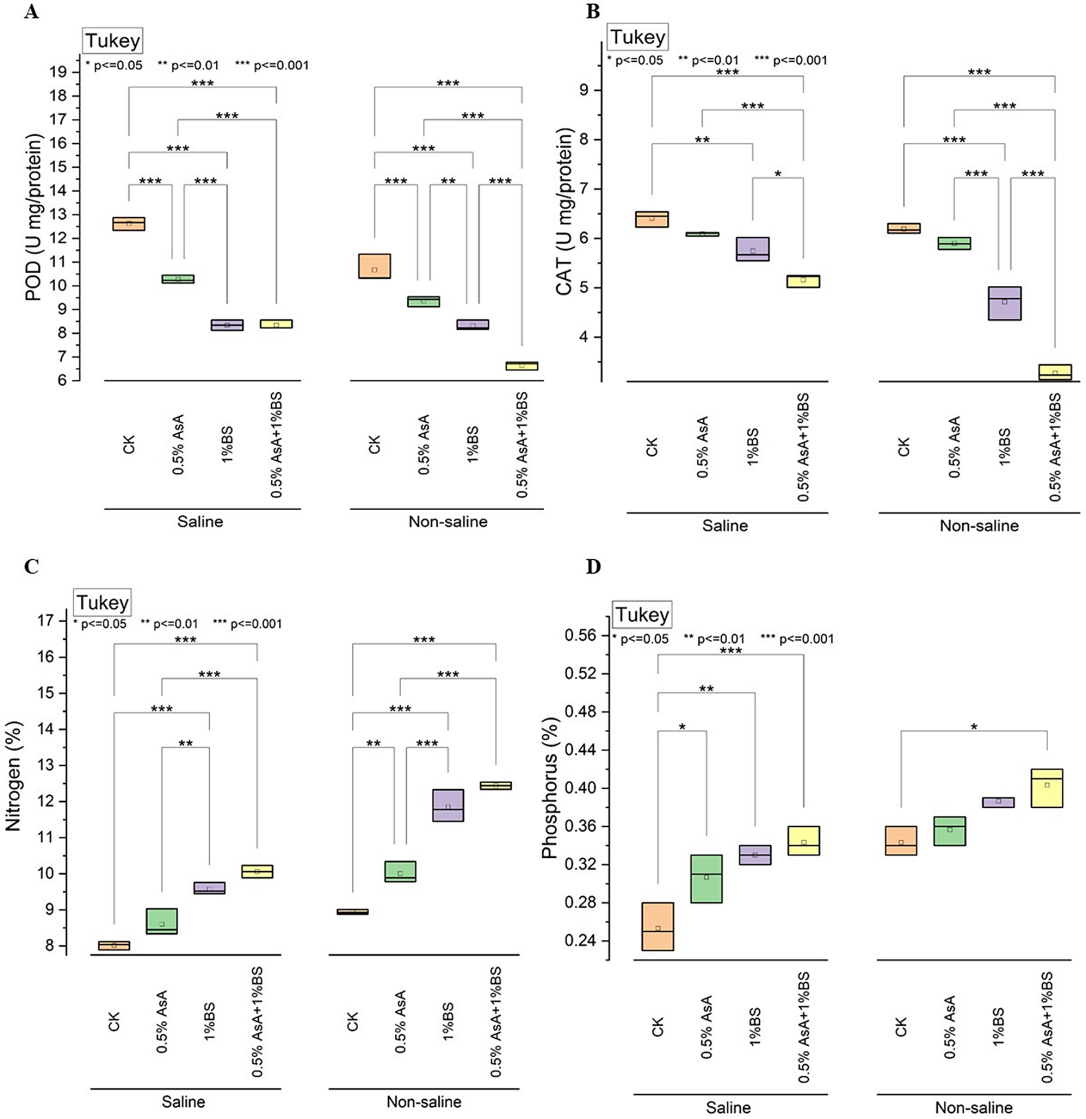
Effect of applied treatments of ascorbic acid (AsA) and sulphur treated biochar (BS) on the peroxidase (**POD**) (**A**), CAT Umg/protein (**B**), Nitrogen(%) (**C**), Phosphorus(%) (**D**) of tomato plants under saline and non-saline growth conditions. The asterisk (*) on the boxplot shows the significant difference among the treatments based on Tukey’s HSD test at *p* ≤ 0.05, 0.01, and 0.001 level

Investigating Peroxidase (POD) activity, measured in units per milligram of protein, in tomato plants grown under varying conditions and subjected to different treatments revealed notable trends. Under saline conditions, the control (CK) exhibited a POD activity of 12.63 units/mg protein. Applying 0.5% Ascorbic Acid (AsA) decreased POD activity to 10.27 units/mg protein. Applying 1% Sulfur-treated Biochar (BS) resulted in a further reduction, measuring 8.34 units/mg protein. Remarkably, the combination treatment of 0.5% AsA with 1% BS also exhibited a POD activity of 8.34 units/mg protein, indicating a similar effect to the standalone BS treatment (Fig. 3A). In contrast, under non-saline conditions, CK displayed a POD activity of 10.67 units/mg protein. Application of 0.5% AsA led to a slight decrease to 9.36 units/mg protein, while 1% BS resulted in a further reduction to 8.32 units/mg protein. Notably, the combination treatment of 0.5% AsA with 1% BS exhibited the most substantial decrease in POD activity, measuring 6.65 units/mg protein compared to CK.

Investigation into Catalase (CAT) activity, measured in units per milligram of protein, revealed significant trends. Under saline conditions, CK exhibited a CAT activity of 6.41 units/mg protein. Applying 0.5%, AsA resulted in a slight decrease in the protein content to 6.09 units/mg. Similarly, using 1% BS led to a further reduction to 5.75 units/mg protein. Remarkably, the combination treatment of 0.5% AsA with 1% BS displayed the lowest CAT activity, measuring 5.16 units/mg protein (Fig. 3B). In contrast, the CK group exhibited a CAT activity of 6.19 units/mg protein under non-saline conditions. Applying 0.5%, AsA led to a slight decrease of 5.90 units/mg of protein. Applying 1% BS resulted in a further reduction to 4.72 units/mg protein. Notably, the combination treatment of 0.5% AsA with 1% BS exhibited the most substantial decrease, measuring 3.27 units/mg protein compared to CK.

In examining tomato plants’ Nitrogen content (N%), notable trends emerged under both saline and non-saline conditions. Under saline conditions, applying 0.5%, AsA led to a modest increase in N% by approximately 7.48% compared to CK. In comparison, the application of 1% BS resulted in a more substantial rise of about 19.95%. Impressively, the combination treatment of 0.5% AsA with 1% BS exhibited the highest increase in N% by approximately 25.06% compared to CK (Fig. 3C). Conversely, under non-saline conditions, the individual application of 0.5% AsA led to a considerable increase in N% by approximately 12.07% compared to CK. In comparison, the application of 1% BS resulted in a more pronounced rise of about 32.61%. Remarkably, the combination treatment of 0.5% AsA with 1% BS displayed the most significant enhancement in N% by approximately 39.78% compared to CK.

In analyzing tomato plants’ Phosphorus content (P%), distinct patterns emerged under saline and non-saline conditions. Under saline conditions, applying 0.5%, AsA led to a moderate increase in P by approximately 24% compared to CK. In comparison, the application of 1% BS resulted in a slightly higher rise of about 32%. Notably, the combination treatment of 0.5% AsA with 1% BS displayed the highest increase in P by approximately 36% compared to CK (Fig. 3D). In contrast, under non-saline conditions, the individual application of 0.5% AsA resulted in a noticeable increase in P by approximately 6% compared to CK. In comparison, applying 1% BS led to a more pronounced rise of about 15%. Remarkably, the combination treatment of 0.5% AsA with 1% BS exhibited the most significant enhancement in P by approximately 18% compared to CK.

### Potassium

**Fig. 4:**
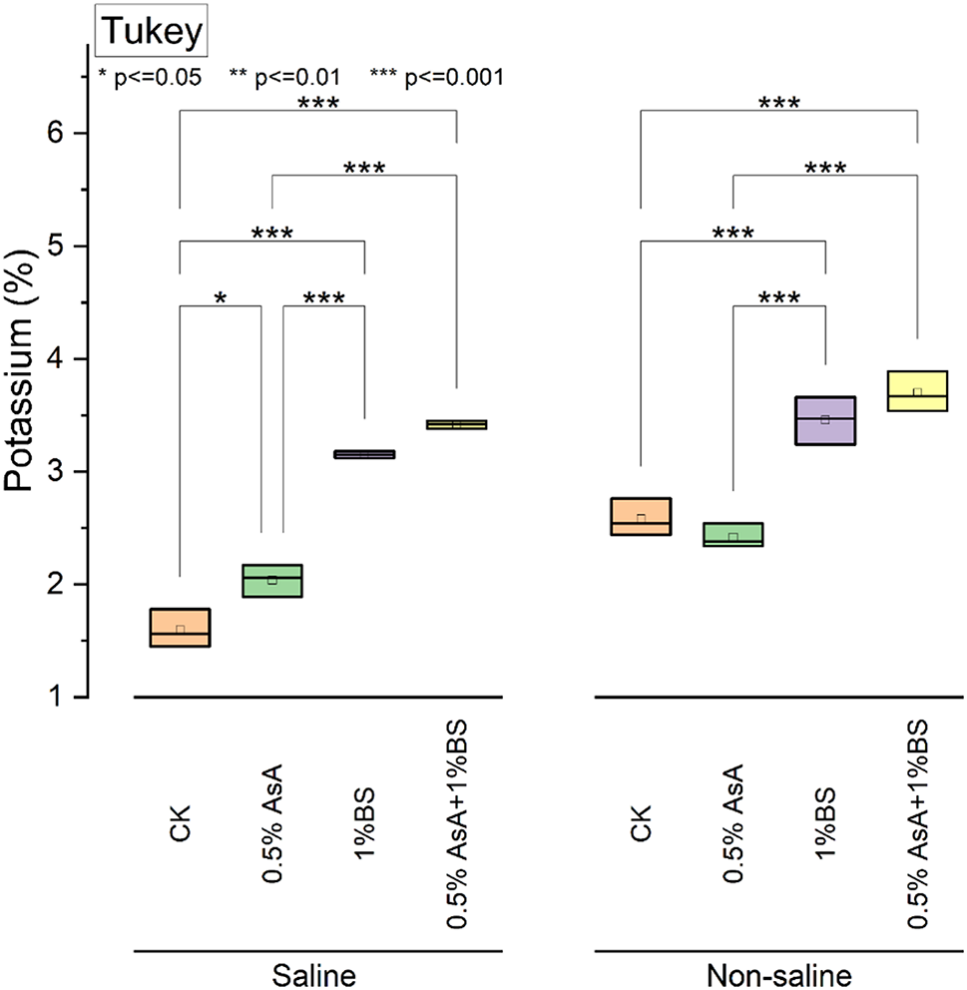
Effect of applied treatments of ascorbic acid (AsA) and sulfur-treated biochar (BS) on the shoot potassium (%) of tomato plants under saline and non-saline growth conditions. The asterisk (*) on the boxplot shows the significant difference among the treatments based on Tukey’s HSD test at *p* ≤ 0.05, 0.01, and 0.001 level

Analyzing tomato plants’ potassium content (K%) under both saline and non-saline conditions relative to the CK group reveals significant variations. Under saline conditions, applying 0.5%, AsA led to a notable increase in K by approximately 27.5% compared to CK. In comparison, the application of 1% BS resulted in a more substantial rise of about 96.88%. Remarkably, the combination treatment of 0.5% AsA with 1% BS exhibited the highest increase in K by approximately 113.75% compared to the CK. Conversely, under non-saline conditions, the individual application of 0.5% AsA decreased K by approximately 6.20% compared to the CK. Applying 1%, BS led to a notable increase of about 34.88%. Notably, the combination treatment of 0.5% AsA with 1% BS exhibited the most substantial enhancement in K by approximately 43.02% compared to the CK (Fig. 4).

## Discussion

The study examined how different doses of ascorbic acid (AsA) mixed with biochar affect the emergence, early seedling growth, and physiological characteristics of tomato plants under salinity stress. We discovered that the simultaneous use of AsA and BC at the right concentration improved salt tolerance in tomato plants by boosting emergence, seedling growth, and physiological functions under salt stress. Seedling growth, namely root and shoot development, is typically robust and establishes a strong basis for achieving optimal crop density, particularly in high salinity conditions [9, 10]. Insufficient seedling emergence is a primary factor that hinders crop establishment in saline conditions. Ascorbic acid (AsA) enhances osmotic balance by improving water retention and root osmotic adjustment, helping plants maintain turgor pressure under saline stress. Additionally, AsA acts as a potent antioxidant, scavenging reactive oxygen species (ROS) and boosting the activity of antioxidant enzymes like superoxide dismutase (SOD), peroxidase (POD), and catalase (CAT) [23–25]. This reduction in oxidative stress protects cellular structures and supports overall plant health. Furthermore, sulphur-treated biochar (BS) enhances cation exchange capacity (CEC), improving the soil’s retention of essential nutrients while limiting sodium uptake [24]. This increase in nutrient availability facilitates better growth performance, allowing plants to effectively absorb vital macronutrients such as nitrogen, phosphorus, and potassium.

Dissolved salts in the soil solution near plant roots might hinder plant growth. The osmotic impact reduces the water uptake of plants, leading to a decrease in the water potential of the plant’s leaves and tissues [11, 12]. An elevated accumulation of salts in the plant’s tissues will hinder the plant’s growth and productivity by disrupting crucial processes such as germination, photosynthesis, nutrient balance, and redox balance. This will restrict the plant from achieving its maximum growth potential [13, 14].

Research has demonstrated that biochar can trap sodium ions, restricting plant roots’ absorption. Our study shows that raising the biochar quantity to 2 tons per hectare successfully counteracts the decrease in fruit yield due to salinity and reduces the sodium accumulation in the roots and fruit. Xiao et al. (2022) found a reduction of salt levels in wheat grains grown in low-fertile soil affected by salinity in the Yellow River Delta by adding 12 tons of biochar per hectare [15, 16]. Increasing the amount of biochar added to a tomato plant could enhance its ability to adapt to saline water by decreasing the absorption of sodium into the plant tissue. However, this aspect was not explored in this study. This theory was never tested. Extreme caution is necessary when adding biochar to clay soil as a high amount could upset the balance between liquid and gaseous phases, reducing the water available in the rhizosphere [17, 26].

The combined treatment of 0.5% AsA with 1% BS exhibited the most significant enhancement, showing a 26.73% increase compared to the CK. In contrast, the percent increase in non-saline conditions compared to the CK group was lower for all treatments. Treatment with 0.5% AsA resulted in a modest 3.45% increase, while 1% BS led to a more noticeable 10.15% increase in fruit diameter. Interestingly, the application of 0.5% AsA and 1% BS showed slight decreases in TSS levels compared to the CK, with values of 8.25 °Brix and 8.34 °Brix, respectively. Notably, the combination treatment of 0.5% AsA with 1% BS resulted in a more pronounced decrease, with a TSS level of 7.56 °Brix. In contrast, the CK displayed a TSS level of 8.34 °Brix under non-saline conditions.

Superoxide dismutase (SOD) is a primary scavenger of O2 – and its enzymatic activity leads to the formation of H2O2 and O2. H_2_O_2_ is eliminated by peroxidases (POD) or catalases (CAT) into water and oxygen [18, 27, 28]. Salt stress led to a notable decrease in the activity of POD, SOD, and CAT enzymes in our research. Another study confirmed that under salt stress, the activities of POD, SOD, and CAT were decreased, as observed by [29–32]. Reduced enzyme activity in stressed plants may have triggered the accumulation of H_2_O_2_ in plant cells under stress, ultimately leading to damage in biological systems [33, 34]. Contrary findings were presented by [29, 35], indicating that salt stress led to elevated activity of POD, SOD, and CAT. Elevating antioxidant enzymes during salt stress may indicate a rise in reactive oxygen species (ROS) and an enhancement of a defense mechanism to reduce oxidative damage caused by plant stress [36, 37]. 200 millimolar NaCl POD, SOD, and CAT showed the highest activity at a high level of AsA and the lowest activity at 0 0.5%level of AsA. Notably, the combination treatment of 0.5% AsA with 1% BS showed the most significant reduction in SOD activity, recording 19.41 units/mg protein [38–40]. Biochar application in salty soil can enhance seedling growth physiological and biochemical processes through the controlled release, retention, or immobilization of nutrients due to its surface features, such as cation exchange and anion exchange capacity [30, 35, 41]. The study revealed that the use of BC elevated the activity of antioxidant enzymes, including CAT, SOD, and POD, in sorghum seedlings. Many researchers have found that the administration of BC improves stress tolerance by enhancing the activity of POD, SOD, and CAT. The BC treatment can control the production of antioxidant enzymes in plants, improving their tolerance to salt stress. However, other studies have shown that the application of BC decreased the activity of the POD, POD, and CAT enzymes in salt conditions; the combination treatment of 0.5% AsA with 1% BS displayed a CAT activity of 5.16 units/mg protein. The decrease in antioxidant enzyme activity may be linked to the ability of biochar to immobilize heavy metals, resulting in reduced metal translocation into plant tissues [31, 42–44]. The most significant levels of POD, SOD, and CAT activity were found when using a 4% BC treatment. The activity of antioxidant enzymes rose as the concentration of ASA increased under salt-stress conditions [45–48]. Our investigation found that external ASA might enhance the activity of POD, SOD, and CAT in the presence of NaCl. ASA was found to strengthen the activities of POD, SOD, and CAT, which are essential for the anti-oxidative defense needed by plants under saline conditions [19, 20, 49, 50]. Enhanced activity of antioxidant enzymes may result from the regenerative properties of ascorbate, which aids in neutralizing reactive forms of molecular oxygen by direct action or enzymatic processes [51–53].

**Fig. 5:**
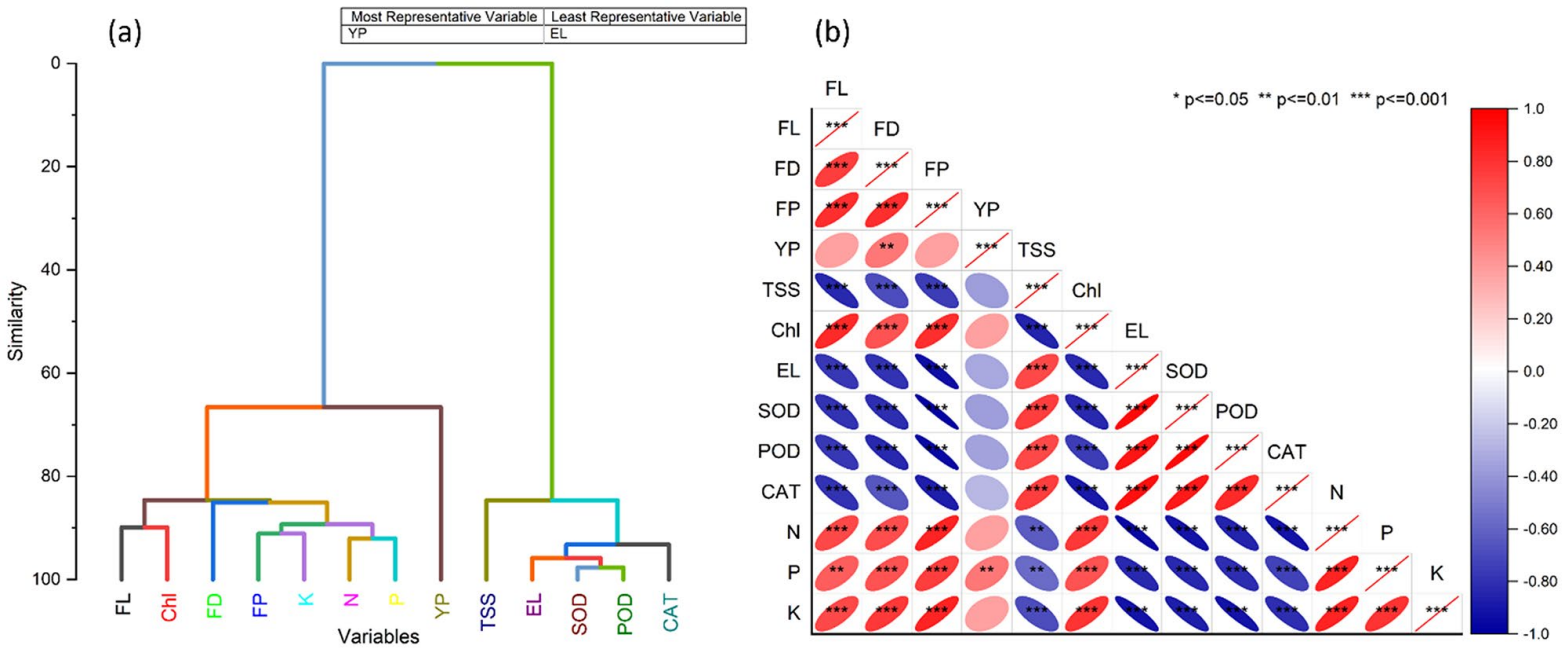
Hierarchical cluster analysis (**a**) and Pear correlation (**b**) among the studied tomato characteristics. Where FL = fruit length, Chl = chlorophyll, FD = fruit diameter, FP = fruit per plant, K = potassium, N = nitrogen, P = phosphorus, YP = yield per pot, TSS = total soluble solids, EL = electrolyte leakage, SOD = superoxidase dismutase, POD = peroxidase, CAT = catalase

The study’s findings are expected to provide valuable insights into the effectiveness of ascorbic acid and sulfur-treated biochar in promoting tomato resilience under saline stress. Positive outcomes, such as improved plant growth, yield, and physiological parameters, would indicate the potential utility of these interventions in mitigating the adverse effects of saline soil conditions on tomato cultivation(Fig. 5). Moreover, the study may highlight the importance of tailored management practices for different environmental conditions to enhance crop resilience and ensure food security [38, 48, 54].

In addition, the acid-modified biochar had a stronger beneficial impact on the plant’s dry matter quantity and yield at the same addition level compared to regular biochar in this experiment. ascorbic acid was likely used to enhance the physicochemical qualities of biochar by removing metals and contaminants from its surface and pores, increasing its pore structure and specific surface area [21, 22, 55]. Acid-modified biochar has enhanced nutrient absorption capacity due to its larger and more numerous internal pores, leading to gradual nutrient release throughout plant growth and minimizing soil nutrient depletion. Biochar is typically alkaline; however, treating it with phosphoric acid lowers its pH [56–58]. Acid-modified biochar added to saline soils neutralizes alkalinity and regulates soil pH more effectively. The effectiveness of this biochar is attributed to several key physical and chemical properties. Firstly, its high surface area and porosity enhance its ability to retain water and nutrients, providing better root zone conditions for the plants. Secondly, the biochar’s cation exchange capacity (CEC) is significantly improved due to the sulfur treatment, which increases its ability to retain essential nutrients like potassium (K⁺), calcium (Ca²⁺), and magnesium (Mg²⁺), while also reducing sodium (Na⁺) uptake under saline conditions [28, 59]. Furthermore, sulfur modification enhances the biochar’s chemical reactivity, helping to improve nutrient availability and reduce soil salinity [60]. These specific characteristics surface area, porosity, and enhanced CEC are crucial in explaining the biochar’s effectiveness in improving plant growth and resilience under salinity stress.

### Pearson correlation analysis

The pearson correlation analysis presented a relatively strong association between multiple variables in the dataset. Plant dry weight showed a very strong positive correlation of 1 with plant height, meaning increased plant height led to a significant increase in plant dry weight. Similarly, No. of primary branches/plant, Fruit length, fruit girth, fruit yield, chlorophyll a, and chlorophyll b indicated positive relations with almost 1 value. However, factors such as EL, %, TSS, MDA, POD, CAT, and SOD had a strong negative correlation, which can be deemed as a negative relation with other variables. But surprisingly, leaves N, leaves P, leaves K, and leaves Na presented correlations. Leaves N and P expressed an almost positive correlation of 0.985, and leaves K and Na had positive and negative correlations of around 0.997 and − 0.986, which suggests the association among the following variables. In the HCA (Fig. 5a), traits such as fruit length (FL), fruit diameter (FD), and yield per pot (YP) clustered closely, indicating strong interrelationships. Similarly, in the Pearson correlation matrix (Fig. 5b), positive correlations were observed between chlorophyll content (Chl) and enzymatic activities like SOD, POD, and CAT, suggesting a coordinated response to stress conditions. In contrast, electrolyte leakage (EL) showed a negative correlation with these parameters, highlighting its role as a stress marker. We’ve also clearly labeled each characteristic (FL, Chl, FD, etc.) in the figure for ease of interpretation. These clarifications provide a clearer understanding of how tomato characteristics interact under different treatments.

## Conclusion

The findings of this research emphasize the potential of 0.5% Ascorbic Acid (AsA) and 1% Sulphur-treated Biochar (BS) to enhance tomato crop resilience under both saline and non-saline conditions. While these treatments demonstrated significant improvements in fruit yield and quality, as well as physiological responses, their scalability in commercial production is crucial to consider. The effective application of these treatments can improve crop productivity, but challenges such as cost, availability of biochar, and optimal application rates must be addressed for widespread adoption.The combined use of AsA and BS not only improved fruit morphology and yield but also enhanced nutrient uptake, suggesting a beneficial synergy that could be leveraged in sustainable farming practices. However, limitations such as the controlled environment of the study highlight the need for further research in field settings to validate these results under varying conditions. Future investigations should focus on optimizing application protocols, assessing the economic feasibility of large-scale implementation, and exploring the long-term environmental impacts of integrating these treatments into conventional tomato production systems. By addressing these aspects, we can better understand the practical implications of these findings and their role in enhancing tomato crop resilience to environmental challenges.

## Data Availability

All data generated or analysed during this study are included in this published article.

## Declarations

### Experimental research and field studies on plants (either cultivated or wild), including the collection of plant material, must comply with relevant institutional, national, and international guidelines and legislation

We confirmed that all methods were performed in accordance with the relevant guidelines / regulations / legislation.
